# Sustained-release voriconazole-thermogel for subconjunctival injection in horses: ocular toxicity and in-vivo studies

**DOI:** 10.1186/s12917-020-02331-5

**Published:** 2020-04-16

**Authors:** Mariano Mora-Pereira, Eva M. Abarca, Sue Duran, William Ravis, Richard J. McMullen, Britta M. Fischer, Yann-Huei Phillip Lee, Anne A. Wooldridge

**Affiliations:** 1 J. T. Vaughan Large Animal Teaching Hospital, Auburn University, Auburn, AL USA; 2 Department of Drug Discovery and Development, Auburn University, Auburn, AL USA

**Keywords:** Keratomycosis, Voriconazole, Triazole, Equine, Periocular, Hydrogel, Fungal, Keratitis

## Abstract

**Background:**

Keratomycosis is a relatively common, sight threatening condition in horses, where treatment is often prolonged and costly. Subconjunctival (SCo) injections offer less resistance to drug diffusion than the topical route, resulting in better penetration to the ocular anterior segment. Voriconazole, a second generation triazole antifungal, is effective against common fungal organisms causing keratomycosis. If combined with a thermogel biomaterial, voriconazole can be easily injected in the SCo space to provide sustained drug release. The purpose of this study was to evaluate the drug concentrations in the anterior segment and clinical effects after SCo injections of voriconazole-containing thermogel: poly (DL-lactide-co-glycolide-b-ethylene glycol-b-DL-lactide-co-glycolide) (PLGA-PEG-PLGA) in healthy equine eyes.

**Results:**

Voriconazole aqueous humor (AH) and tear concentrations were compared between 6 horses, receiving 1% voriconazole applied topically (0.2 mL, q4h) (Vori-Top) or 1.7% voriconazole-thermogel (0.3 mL) injected SCo (Vori-Gel). For the Vori-Gel group, voriconazole concentrations were measured in AH and tears at day 2 and then weekly for 23 days, and at day 2 only for the Vori-Top group. Ocular inflammation was assessed weekly (Vori-Gel) using the modified Hackett-McDonald scoring system. Ocular tissue concentrations of voriconazole following SCo 1.7% voriconazole-thermogel (0.3 mL) injections were evaluated post euthanasia in 6 additional horses at 3 different time points. Three horses received bilateral injections at 2 h (*n* = 3, right eye (OD)) and 48 h (*n* = 3, left eye (OS)) prior to euthanasia, and 3 horses were injected unilaterally (OS), 7 days prior to euthanasia. Voriconazole-thermogel was easily injected and well tolerated in all cases, with no major adverse effects. On day 2, drug concentrations in tears were higher in the Vori-Top, but not statistically different from Vori-Gel groups. For the Vori-Gel group, voriconazole was non-quantifiable in the AH at any time point. Total voriconazole concentrations in the cornea were above 0.5 μg/g (the target minimum inhibitory concentration (MIC) for *Aspergillus* sp.) for up to 48 h; however, concentrations were below this MIC at 7 days post treatment.

**Conclusions:**

Voriconazole-thermogel was easily and safely administered to horses, and provided 48 h of sustained release of voriconazole into the cornea. This drug delivery system warrants further clinical evaluation.

## Background

Fungal keratitis is a condition that affects both humans and animals, where prolonged, intensive treatment and relatively poor visual outcome may result in considerable medical and economic implications [1–3]. Horses are more susceptible to corneal fungal infections secondary to ocular trauma. This may be due to their globe position, their behavior, or to other environmental factors that may be more common in horses compared with other animals, thus making the equine species a natural model for human keratomycosis [2, 4, 5]. Drug delivery to the eye is labor intensive in horses due to their size and behavior, and ocular barriers can prevent drugs from reaching the target ocular tissue [6, 7]. To overcome this, frequent instillations of medications are required, with potential concurrent oral antifungals, subconjunctival (SCo) and/or intrastromal corneal injections [4, 8–11]. In both people and horses, voriconazole, a second-generation triazole, has become the first choice of treatment for keratomycosis. It has a broad spectrum of activity, good corneal penetration, and a low minimum inhibitory concentration (MIC) for common fungi implicated in this disease, such as *Aspergillus* sp. and *Fusarium* sp., when compared to other azole drugs [2, 5, 12–14]. In the ranges of MICs for filamentous and yeast organisms, most isolates are < 0.5 μg/mL, therefore antifungal therapies are expected to have MICs of unbound drug above this value to be consider of clinical efficacy [5, 15–17].

In ocular drug delivery, the goal is to attain sustained therapeutic concentrations of drugs at the target tissue, as well as ease and safety of delivery with minimal intervention [18, 19].

Biomaterials for drug delivery such as thermogels are triblock poly (DL-lactide-co-glycolide-b-ethylene glycol-b-DL-lactide-co-glycolide) (PLGA-PEG-PLGA) copolymers with a three-dimensional network that protect the encapsulated drug from rapid degradation [20]. Thermogels are attractive candidates for targeted drug delivery. These copolymers can be injected in a liquid form, and when it is exposed to body temperature, the solution becomes a solid gel that gradually releases the encapsulated drug [20–23].

As described by Cuming et al., voriconazole-containing thermogel, has shown to be easily injected into the dorsal SCo space of equine eyes, forming a well-defined gel deposit [24]. Furthermore, the voriconazole-PLGA-PEG-PLGA thermogel studied achieved a sustained release of voriconazole above the target MIC of 0.5 μg/mL for more than 28 days in vitro [24]. The demonstrated voriconazole sustained-release from the thermogel, together with the ease of administration in the SCo space, makes this method of potential clinical importance. The use of the voriconazole-thermogel has not been evaluated in live horses prior to this study.

Safety of an ocular drug or a route of administration should be evaluated, and previous methods that induce eye irritation such as the Draize test have been considered problematic to animal welfare [25]. To avoid this, safety can be evaluated in vitro with live/dead cell studies in cell culture, or specifically for corneal irritants, by the bovine corneal opacity and permeability test [26, 27]. Histological analysis alone, whenever euthanasia is the endpoint, is a valuable tool to assess tissue damage due to a drug [28]. In live animals, non-invasive, semiquantitative systems described by McDonald and Shadduck, and Hackett and McDonald are frequently cited in preclinical drug development works [29]. More specifically, slit lamp-based scoring systems are recorded using the modified Hackett-McDonald system, where clinical findings can be semiquantitatively assessed and scores used for further analysis [30].

The objectives of the study were first to evaluate the acute ocular toxicity of SCo injection of voriconazole-thermogel in horses using an ocular inflammatory scoring system and histological analysis. Secondly, to determine the voriconazole concentrations in tear film, aqueous humor (AH), and ocular tissues at different timepoints following a SCo injection of voriconazole-thermogel in the dorsal bulbar conjunctiva. Furthermore, the effect of location (anterior and posterior segments) and site of injection in drug distribution were analyzed.

## Results

### Clinical findings and ocular toxicity

**Fig. 1:**
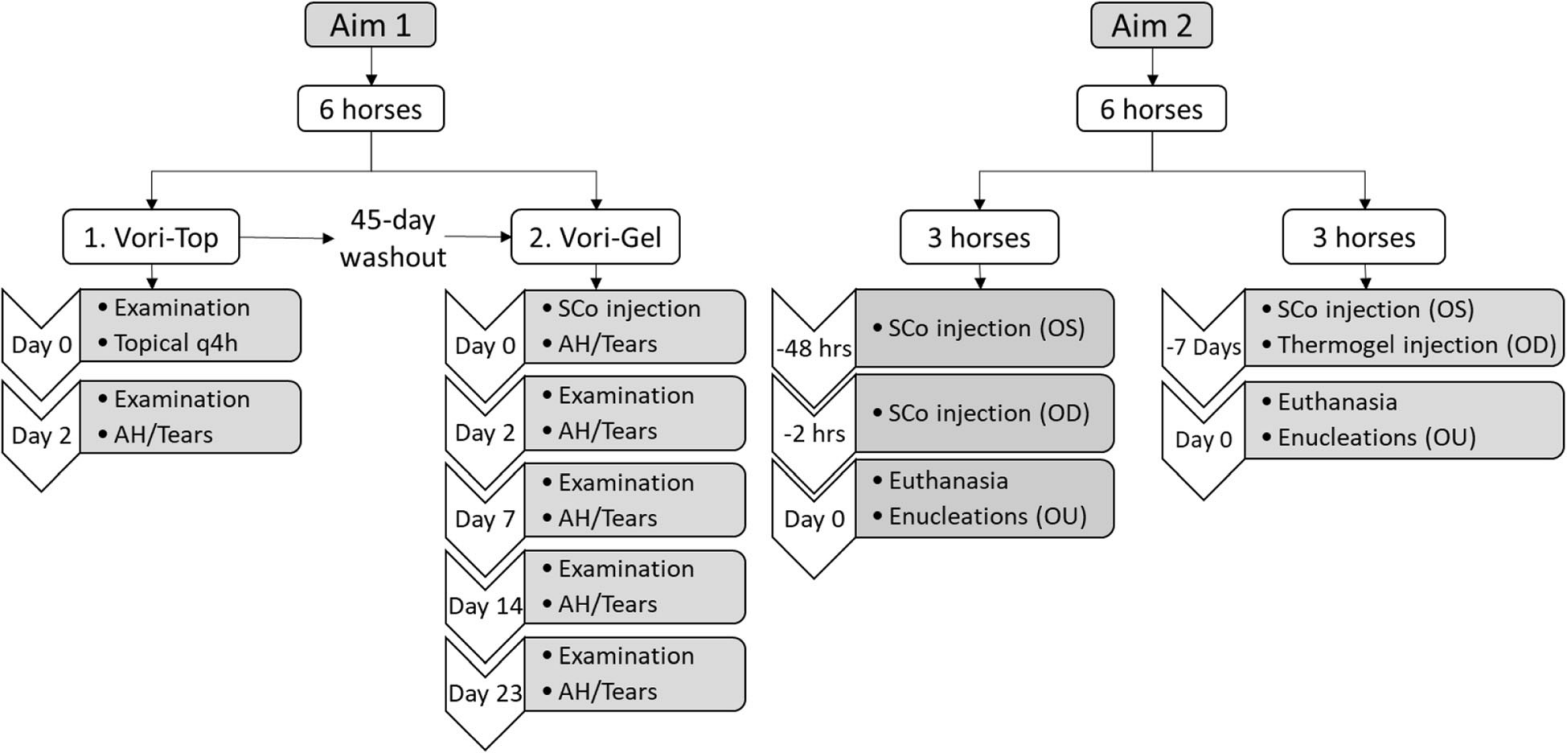
Flow chart of the experimental design describing both aims of the study. AH, aqueous humor; SCo, subconjunctival; OS, left eye; OD, right eye; OU, both eyes. Topical refers to the instillation of 1% voriconazole solution; injection refers to the SCo voriconazole thermogel

**Fig. 2:**
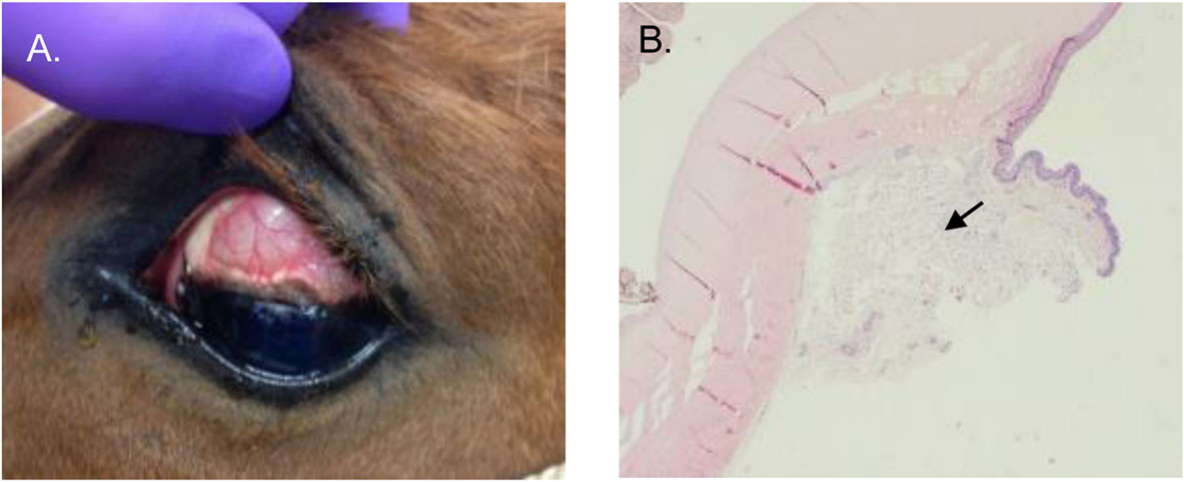
**a** Gel deposit in the dorsal bulbar subconjunctival space immediately after injection of the voriconazole-thermogel. **b** Histology of the eye globe 7 days post thermogel injection in 1 horse; the arrow indicates the injection site with macrophage infiltration and lipid-like material

As part of the inclusion criteria, results from complete blood count (CBC) and serum biochemical analysis (SBA) were within normal values for all the 12 horses prior to enrollment in each part of the study (the study design is described in Fig. 1). A baseline complete ophthalmic examination, performed by a board certified veterinary ophthalmologist (EMA or RJM), was normal for all the horses. All 12 horses tolerated the SCo injections and had normal physical examinations with no evidence of ocular pain for up to 23 days (total duration of the study). The 0.3 mL volume of the 1.7% voriconazole-thermogel was easily delivered in the SCo space using a 30 gauge needle as described [24]. After injection, a well-defined gel deposit was observed in the dorsal bulbar conjunctiva (Fig. 2a).

Histological analysis performed in 2 globes harvested 7 days after SCo injection of the thermogel alone did not show any significant structural abnormalities related to the thermogel. In 1 horse, mild macrophagic inflammatory reaction in the conjunctival substantia propria, along with intra-histiocytic and free lipid material was observed. (Fig. 2b).

**Fig. 3:**
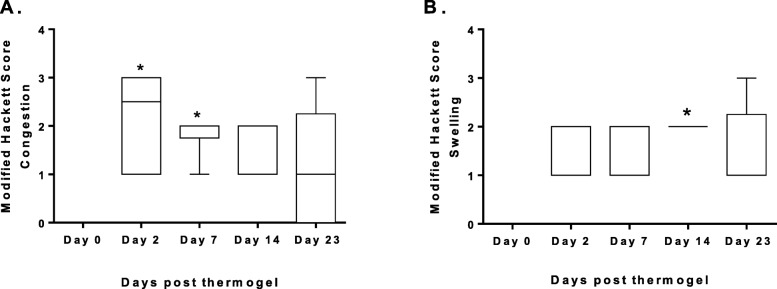
Modified Hackett-McDonald score for conjunctival inflammation at timepoints post voriconazole thermogel injection. Data are presented as box and whiskers where the line is the median, the box represents the interquartile range, and the whiskers are minimum and maximum. **a** Conjunctival congestion; *indicates days 2 and 7 are significantly higher than day 0 (*p* = 0.0053). **b** Conjunctival swelling; *indicates day 14 is significantly greater than day 0 (*p* = 0.0021)

Results from the evaluation of conjunctival congestion and swelling from the 6 horses receiving a SCo injection of voriconazole thermogel (Vori-Gel) as part of the first study aim are presented (Fig. 3). There was significantly more swelling on day 14 when compared to day 0 (*p* = 0.0021). Conjunctival congestion on days 2 and 7 post treatment with the voriconazole-thermogel were significantly higher than day 0 (*p* = 0.0053). There were no significant differences between days for conjunctival discharge in any of the horses (*p* = 0.2311).

Scores for corneal cloudiness, corneal neovascularization, fluorescein stain uptake, aqueous flare, and iris congestion were 0 at all evaluation time points throughout the study. No observable changes were identified in the anterior vitreous, lenses or fundi at all evaluation time points on any horses throughout the study.

### Voriconazole concentrations in aqueous humor and tears

For the 6 horses included in the first aim of the study (Fig. 1), voriconazole concentrations in tears on day 2 (1 h after treatment in the Vori-Top group and 2 days after SCo injection in the Vori-Gel group) were higher, however not significantly different (*p* = 0.0625), in the Vori-Top group compared with the Vori-Gel. Following the SCo injection, voriconazole concentrations in tears were not detectable (< 0.001 μg/mL) on days 7, 14 and 23. In AH, on days 2 and 7 post injection, voriconazole was detectable but non-quantifiable (concentrations < 0.005 but more than 0.001 μg/mL), and not detectable (< 0.001 μg/mL) on days 14 and 23 post injection. Voriconazole concentrations in AH and tears for the Vori-Top and Vori-Gel groups are presented in Table 1.

**Table 1:** Voriconazole concentration in tears and aqueous humor for the voriconazole thermogel (Vori-Gel) and topical voriconazole (Vori-Top) groups, *n* = 6

	Day	Mean (μg/mL)	SD	Median (μg/mL)	Range
**Vori-Gel (1.7% voriconazole thermogel)**					
Tears	2	0.15	0.21	0.05	0.02–0.19
	7	0	0	0	0–0.02
	14	0	0	0	0–0
	23	0	0	0	0–0
Aqueous humor	2	0.001–0.005	0.001–0.005	0.003	0.001–0.005
	7	0.001–0.005	0.001–0.005	0.003	0.001–0.005
	14	0	0	0	0–0
	23	0	0	0	0–0
**Vori-Top (1% voriconazole solution)**					
Tears	2	3.50	3.18	3.04	0.96–8.95
Aqueous humor	2	1.52	0.31	1.42	1.25–2.07

### Voriconazole ocular tissue concentrations and distribution

**Fig. 4:**
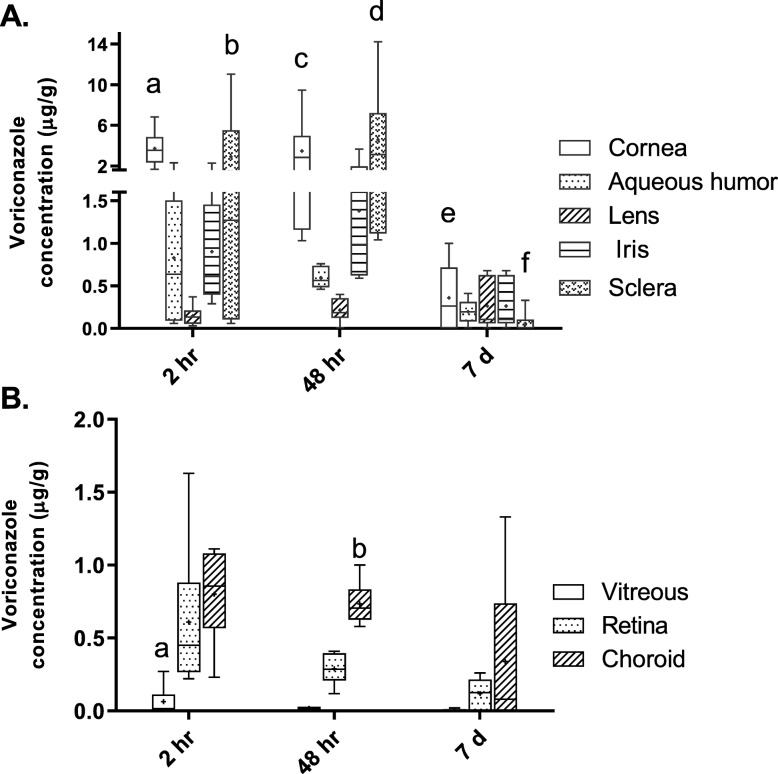
Voriconazole concentrations of individual tissues 2 h, 48 h, and 7 d after thermogel injection. All data are presented as box and whisker plots where the box is the interquartile range, the whiskers are the minimum and maximum, the line is the median, and the + sign is the mean. **a** Concentrations of voriconazole in tissues of the anterior segment. The ciliary body was combined with the iris for analysis. Note the split y-axis scale so that the full range of concentrations can be seen. The letters indicate significant differences (*P* < 0.05); a = cornea greater than aqueous humor (AH), lens, and iris at 2 h; b = sclera greater than lens at 2 h; c = cornea greater than AH and lens at 48 h; d = sclera greater than AH, lens, and iris at 48 h; e = cornea at 7 d less than cornea at 2 h and 48 h; f = sclera at 7d less than sclera at 2 h and 48 h. **b** Concentrations of voriconazole in tissues of the posterior segment. The letters indicate significant differences (*P* < 0.05); a = vitreous less than retina and choroid at 2 h; b = choroid greater than vitreous and retina at 48 h

Following voriconazole-thermogel SCo injection in the 6 horses included in the second aim of the study (Fig. 1), total voriconazole concentrations in cornea were significantly higher than in AH and lens (p < 0.0001) at both the 2 h and 48 h timepoints. No statistical differences between voriconazole concentrations in tissues of the anterior segment were observed within the 7 days timepoint. Moreover, at the 48 h timepoint, the mean and minimum range of voriconazole concentrations in iris-ciliary body and sclera, were consistently above the target MIC of 0.5 μg/g. Most importantly, total voriconazole concentrations in cornea at both 2 and 48 h maintained minimum concentrations above the target MIC (Fig. 4a). Table 2 shows the mean (+/− SD), medians and ranges of ocular tissue voriconazole concentrations for the 6 horses included at the different timepoints.

**Table 2:** Voriconazole concentrations in the different ocular tissues for the 3 timepoints. The data for the 2 h and 48 h timepoints were from opposite eyes from the same 3 horses

		2 h (***n*** = 3)		48 h (***n*** = 3)		7 days (***n*** = 3)	
		Mean/Median (μg/g)	SD/Range	Mean/Median (μg/g)	SD/Range	Mean/Median (μg/g)	SD/Range
**Cornea**	Dorsal	4.43/3.90	2.17/2.58–6.82	5.04/3.15	3.83/2.52–9.45	0.33/0.00	0.33/0.00–1.00
	Ventral	3.01/3.19	1.28/1.65–4.19	1.91/1.20	1.37/1.03–3.49	0.38/0.41	0.14/0.12–0.62
**Aqueous humor**	Dorsal	0.14/0.10	0.03/0.06–0.15	0.61/0.61	0.15/0.46–0.76	0.30/0.28	0.06/0.20–0.41
	Ventral	1.56/1.23	0.66/1.12–2.32	0.58/0.52	0.13/0.49–0.73	0.10/0.11	0.06/0.00–0.19
**Lens**	Dorsal	0.19/0.16	0.17/0.03–0.37	0.30/0.34	0.12/0.16–0.40	0.07/0.11	0.04/0.00–0.11
	Ventral	0.11/0.11	0.05/0.06–0.16	0.13/0.18	0.10/0.01–0.19	0.17/0.14	0.04/0.13–0.24
**Iris**	Dorsal	1.41/1.18	0.78/0.78–2.28	2.03/1.41	1.43/1.02–3.67	0.26/0.11	0.21/0.00–0.68
	Ventral	0.39/0.43	0.08/0.29–0.44	0.72/0.63	0.20/0.59–0.95	0.26/0.10	0.17/0.08–0.61
**Sclera**	Dorsal	5.69/3.68	4.68/2.35–11.04	7.35/4.88	6.03/2.95–14.22	0.11/0.00	0.11/0.00–0.33
	Ventral	0.12/0.12	0.07/0.06–0.19	1.83/1.14	1.28/1.04–3.30	0.01/0.00	0.01/0.00–0.03
**Choroid**	Dorsal	0.66/0.68	0.42/0.23–1.07	0.81/0.78	0.18/0.64–1.00	0.50/0.16	0.42/0.00–1.33
	Ventral	0.94/0.99	0.20/0.72–1.11	0.66/0.68	0.08/0.58–0.73	0.18/0.00	0.18/0.00–0.54
**Retina**	Dorsal	0.89/0.63	0.65/0.40–1.63	0.35/0.39	0.08/0.26–0.41	0.15/0.20	0.08/0.00–0.26
	Ventral	0.33/0.28	0.15/0.22–0.50	0.22/0.24	0.10/0.12–0.31	0.08/0.09	0.05/0.00–0.16
**Vitreous**	Dorsal	0.03/0.02	0.03/0.01–0.00	0.02/0.01	0.01/0.01–0.03	0.00/0.00	0.00/0.00–0.01
	Ventral	0.10/0.01	0.15/0.01–0.27	0.02/0.01	0.01/0.01–0.03	0.01/0.01	0.01/0.00–0.02

**Fig. 5:**
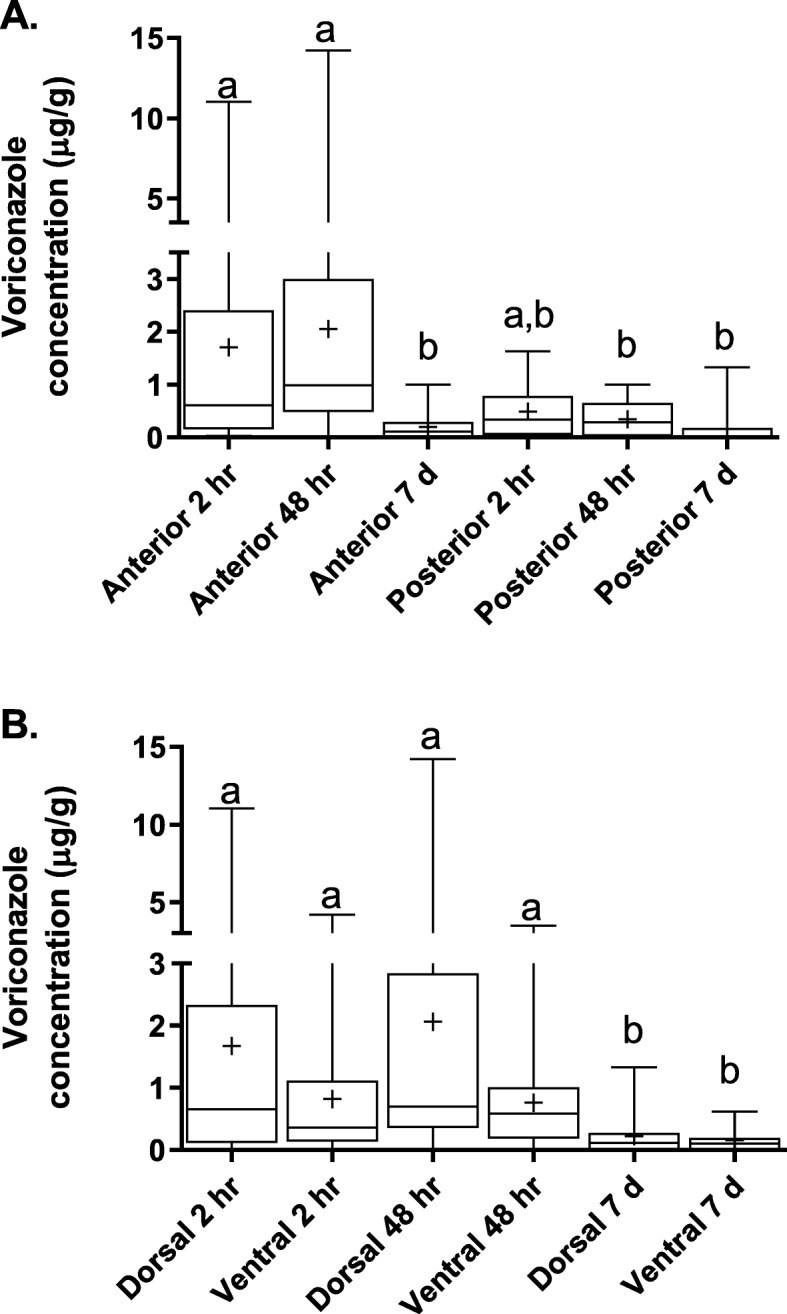
Pooled tissue concentrations of the tissues of the anterior and posterior segments (**a**) and dorsal and ventral sections of the eye (**b**) for each time point. All data are presented as box and whisker plots where the box is the interquartile range, the whiskers are the minimum and maximum, the line is the median, and the + sign is the mean. Note the split y-axis scale so that the full range of concentrations can be seen. Anterior segment contains: cornea, aqueous humor, iris-ciliary body, sclera and lens. Posterior segment contains vitreous, retina and choroid. Different letters within each panel (a and b) are significantly different (*p* < 0.05)

Within the posterior segment of the eye, at 2 h post treatment, voriconazole concentrations were significantly lower in vitreous compared to retina and choroid (*p* < 0.0001). At the 48 h timepoint choroidal concentrations were significantly higher than vitreous and retina (*p* < 0.0001); concentrations in the tissues of the posterior segment at the 7 day timepoint were not significantly different. Only the choroid at the 48 h timepoint had a lower limit of total voriconazole concentrations that consistently exceeded the target MIC for all samples (Fig. 4b). Voriconazole concentrations in the pooled tissues of the anterior segment was significantly higher at the 2 and 48 h timepoints compared to the posterior segment at 48 h, and both anterior and posterior segments at 7 days post treatment (*p* < 0.0001). When all the tissues were pooled for anterior and posterior segments respectively, the minimum concentration was below the target MIC of 0.5 μg/mL; however it is important to observe that at the 48 h timepoint, voriconazole concentrations were above this MIC in 75% of analyzed samples (Fig. 5a).

No significant differences were observed between the grouped dorsal and ventral segments of the eye (*p* = 0.4823) for the 2 and 48 h timepoints. (*p* < 0.0001). However, as shown in Table 2, most of the tissues of the dorsal segment showed a tendency to have higher concentrations. Regarding the cornea, at both 2 and 48 h, concentrations in the ventral segments (opposite from the injection site) were above the MIC of 0.5 μg/mL. No significant effect of the injection, performed in the dorsal bulbar conjunctiva, on location was observed within each timepoint (Fig. 5b).

## Discussion

Our study has shown that following thermogel injection, voriconazole was released to the ocular tissues, especially cornea, for at least 7 days, and signs of ocular pain due to the injection were not noted in any horse at any time point. While results are very promising, when evaluating drug concentrations in tissues, there are considerations. Protein binding was not measured in the tissue homogenates, however it is expected to be comparable to the 31.68 ± 1.92% reported in plasma for horses [31–33]. Even though we could not determine the voriconazole distribution on a cellular level, the various compartments from the tissue samples are potential targets of fungal invasion [34]. Based on the drug concentration ranges observed for the dorsal and ventral cornea (1.03–9.45 μg/g), if protein binding is similar to serum, voriconazole is expected to be above the target MIC for up to 48 h, which is of significance for the treatment of keratomycosis [35, 36]. The maintenance of voriconazole concentrations in ranges from 1.04–14.22 μg/g in the sclera is of importance, since transcleral drug diffusion is very efficient in allowing drug molecules to reach the cornea [37]. Our data showed significant individual variation in voriconazole concentrations, both in tissues as in tears, therefore, in some individuals, therapeutic concentrations might not be reached in tissues other than the cornea. A difference between the voriconazole concentrations in AH obtained after 48 h of the SCo injection between horses of the first aim and the second aim was not expected. This could be due to slight difference in sample processing and analysis, since for horses in the second aim, the frozen AH was processed in the same way as a tissue. During tissue dissection, contamination from another nearby tissue such as cornea or iris was possible, hence contributing to a higher drug concentration.

The SCo injection of voriconazole-thermogel was easily injected in the SCo space and was well tolerated by all the horses, with no evidence of ocular pain observed for up to 23 days. Safety of the technique and behavior of the thermogel were consistent with previous reports of SCo injections in horses and the use of copolymers in ophthalmology [10, 24, 38–41]. Even though there was a significant increase in conjunctival swelling following SCo injection, a score of 2 is considered to be mild [29, 30]. Swelling was most likely an enlargement within the conjunctiva due to the gel deposit, since it was restricted to the area of injection, rather than diffuse edema and inflammation. The further decrease in the median swelling score by day 23, was consistent with the normal degradation of the gel [42]. On days 2 and 7, the conjunctival vascular congestion was most likely due to the injection performed on day 0 or the extraction of AH on day 2. The conjunctival vascular congestion was always confined to a focal area. Hemorrhage following SCo injections is common, and usually self-resolves with no discomfort to the patient [10, 43]. Previous reports of the use of commercial voriconazole sterile injection 1% by SCo injection or intrastromal injections in concentrations of 1 and 5% did not report significant complications, however none of this studies used an inflammatory scoring system for a more objective assessment [9, 10, 44].

It was unlikely that the thermogel alone would have adverse effects, and previous reports using thermogels by SCo injection in rabbits and rats did not show histological abnormalities [45, 46]. Even though only a small number of eyes were analyzed by histology, there were no significant adverse effects in the ocular tissues, and the changes observed in one of the horses was related to the normal degradation process of the copolymer [47]. Moreover, this horse did not show evidence of ocular pain in either eye (thermogel alone or voriconazole-thermogel) for at least 7 days following treatment. Due to the difficulty of having a larger sample size in a large animal model, our data from histology and inflammatory scores, together with evidence from larger studies in other species, suggest that using thermogel for SCo injection is unlikely to produce adverse effects [40, 45, 46].

There is limited literature describing the use of voriconazole by SCo injection in horses. Reports of the use of this route with drugs other than voriconazole have proven to provide therapeutic concentrations to the anterior segment of the eye [48–50]. The topical 1% voriconazole treatment was considered a positive (standard treatment in clinical field) control to validate AH concentrations of voriconazole in this group of horses and as a comparison to previously published results [4]. Even though statistical analysis concluded no difference between concentrations in tears between the Vori-Top and Vori-Gel groups on day 2, from results in Table 1, voriconazole concentrations on day 2 for the Vori-Top group were much higher than the Vori-Gel. Following topical administration, range of voriconazole concentrations in tear film had minimum values above the MIC of 0.5 μg/mL. On the contrary, the maximum voriconazole concentration in tears for the Vori-Gel group was below this MIC. The higher voriconazole concentrations in AH in the Vori-Top group compared with the Vori-Gel group on day 2 could be explained by the higher concentrations in the tear film, since the lipophilic nature of the drug will facilitate transcorneal diffusion to the anterior chamber. In contrast, the voriconazole encapsulated in a thermogel and administered by SCo injection will reach the cornea mainly transclerally and potentially bypassing the anterior chamber [51, 52].

In our study, the minimum concentration of total voriconazole in the target tissue (cornea) at both 2 and 48 h after SCo injection of the voriconazole thermogel was above the target MIC of 0.5 μg/mL. Achieving high concentrations in the target tissue so quickly (by 2 h) after voriconazole-thermogel injection may preclude the need for concurrent topical voriconazole treatment in severe cases of fungal keratitis. In addition, the voriconazole-gel formulation allows a continuous amount of drug to be delivered to the eye from the injection site until the hydrogel has dissipated and all drug is depleted. Continuous infusion of the drug may result in efficacy with a lesser dosage as with intermittent therapy of topical voriconazole. The contribution from crossover of the 5 mg of voriconazole from the SCo injection 48 h prior to euthanasia to the contralateral eye at 2 h was considered negligible based on the estimated percentage of voriconazole that reaches the eye following systemic administration [52–54]. However, the fact that voriconazole plasma concentrations were not measured after SCo injection was a limitation of the study.

The higher concentrations of drug in the sclera demonstrates that the drug will have a sustained release from the site of injection. The sclera was included as a tissue of the anterior segment due to the fact that the SCo injection was applied over the anterior sclera, and this might have contributed to the significance in concentration difference between the anterior and posterior segments. Moreover, 48 h after voriconazole thermogel administration, the minimum concentration in the sclera was about twice the target MIC. This is of importance since the drug accumulated in the SCo space will serve as a reservoir for transcleral drug diffusion to the cornea [36].

Our results showed that most of the drug is accumulated in the ocular anterior segment, the region of interest for treatment of keratomycosis. Higher drug concentrations were expected in the anterior segment compared to the posterior segment, since important mechanisms of drug diffusion to the eye using the SCo route include direct penetration through the sclera and transport via the limbal vessels [36, 52, 55, 56]. Reflux from the injection, as a mechanism of drug diffusion, was deemed to be minimal since a well-defined gel was formed immediately after injection, and a small gauge needle was used [57].

The previously discussed findings are in correlation with other studies of drug diffusion to the eye, were after SCo injection, the drug reaches the anterior segment of the eye, through mechanisms that will highly depend on the drug characteristics (hydrophilic versus lipophilic) [48, 56, 58]. Voriconazole is a lipophilic drug, and this confirms our initial hypothesis in which the drug is expected to accumulate in the tissues. The lower voriconazole concentrations detected in both the frozen ocular fluids, and AH collected by anterior chamber paracentesis compared to tissues could be due to the limited aqueous solubility of the drug [59].

Achieving corneal voriconazole concentrations above the target MIC away from the injection site for up to 48 h is of interest for the treatment of keratomycosis in case the bulbar conjunctiva on the same side as the lesion is unsuitable for injection.

We also demonstrated sustained release of voriconazole from the site of injection for at least 7 days in live horses, however in concentrations below the target MIC of 0.5 μg/mL. Subtherapeutic concentrations leading to treatment failure, as well as long-term therapy, may increase the risk of azole resistance, especially by *Aspergillus fumigatus* [60, 61]. Therefore, therapeutic drug monitoring or further studies are needed for more science-based dosing intervals. For this study, horses without ocular inflammation were used, therefore the diffusion of drugs by the SCo route will be limited by the blood-aqueous barrier, since drug administration via SCo or scleral routes will reach the anterior segment and subsequently the cornea mainly via the iris-ciliary body vessels [51, 52, 62]. High voriconazole concentrations are desired in both the cornea and tear film, and transcorneal diffusion secondary to efflux from the injection site of a lipophilic drug such as voriconazole is expected and independent of ocular inflammation. However, following the SCo route, this is considered a minor contributor to the voriconazole concentrations in the anterior segment of the eye, and the hydrophilic thermogel will enhance the drug diffusion through the blood-aqueous barrier [51, 52].

Minimum concentrations of total voriconazole in the choroid were above the target MIC 48 h following treatment. This could be of interest for further investigation in the treatment of conditions in the posterior segment. Moreover, we demonstrated effective diffusion of a lipophilic drug to the posterior segment of the eye after treatment applied in the anterior segment, which could have been facilitated by the hydrophilic nature of the thermogel [58, 63, 64].

Voriconazole concentrations in the ocular tissues 7 days after SCo injection were below the target MIC in all horses. Based on this result, re-dosing for the voriconazole-thermogel might be necessary in a shorter period of time; however, more research in horses with diseased eyes is needed to better evaluate this therapy. High numbers of individuals are difficult to achieve in a large animal model, however, the data collected in this study may have important clinical implications in other species, since horses serve as a natural model for keratomycosis.

A limitation of this study was the fact that no drug was measured in tissues between days 2 and 7, thus it was not possible to determine for how long concentrations above the target MIC were maintained, limiting the understanding of the concentration-time course. The use of eyes from animals euthanized for different reasons was another limitation regarding the sample size for the voriconazole tissue concentrations phase, since a larger sample size could have given more conclusions for injection location effect. Moreover, availability of animals also limited the inclusion of histological analysis for the 2 and 48 h timepoints, which would have given more support to the safety of the thermogel alone in SCo injections. Measuring drug concentration in tissues rather than fluids represents a challenge, and was another limitation in the number of samples analyzed. The design of the study, where AH samples were taken while evaluating inflammation in the conjunctiva could have been a confounding factor while assessing for vascular congestion, since it was common to have small transient subconjunctival hemorrhages. Regarding tear collection, dilution has to be taken under consideration. The collecting method used may have induced reflex tearing, thus altering sample dilution and contributing to the large variation in drug concentration [51]. Randomization of horses into treatments groups was not done since the time of degradation of the voriconazole-loaded thermogel was unknown. Therefore to avoid carry over of the drug to the other group, it was decided to assign the topical treatment to the first group of horses. Examination for ocular inflammation and collection of AH were done simultaneously in each horse. Since only one board-certified ophthalmologist was available at each time, blinded evaluation by a second ophthalmologist was not possible, which was a limitation of the study.

## Conclusions

This study is the first to use the SCo route for voriconazole delivery from a biodegradable thermogel in live horses. The results of the injection technique were in agreement with a previous report in ex vivo model [24]. Voriconazole-thermogel was easy and safe to administer in horses with no major adverse effects. Voriconazole concentrations in tear film and AH 2 days after Sco injection of the voriconazole thermogel did not reach the target MIC and, although not statistically significant, were lower compared to the topical treatment. However, the thermogel provided sustained release of voriconazole to the cornea and sclera in concentrations above the target MIC for up to 48 h. This information can be translated to the clinical setting to increase treatment efficacy and client compliance.

Further studies are warranted to better determine the frequency of injections, and to better assess the ocular distribution of voriconazole from the thermogel in clinical cases with impaired blood ocular barriers.

## Methods

### Aim 1: evaluation of ocular toxicity and Voriconazole concentrations in aqueous humor and tears

Overall study design is presented in Fig. 1. For the first aim, 6 horses (5 mares and 1 gelding), with an age range of 8–14 and weight range of 460–560 kg were used. Breeds included 4 American Quarter Horses and 2 Tennessee Walking Horses. These horses were returned to their herds at the end of the study.

Using a cross-over design, with no randomization of treatment order, 2 groups of 6 horses each were defined; 1 group was treated topically with 1% voriconazole solution (Vori-Top), and the second group was treated with a SCo injection of 1.7% voriconazole-thermogel (Vori-Gel). The Vori-Top group received a total volume of 2.4 mL of 1% voriconazole solution (0.2 mL q4h) applied directly to the surface of the right eye (OD) using a 1 mL syringe/25-gauge needle hub (needle broken off) combination once every 4 h for 48 h. One hour after the final dose was administered, AH and tears were collected for determination of voriconazole concentrations.

After a 45-day washout period, the same 6 horses formed the Vori-Gel group, which received a single SCo injection of 0.3 mL, 1.7% voriconazole-thermogel in the dorsal bulbar conjunctiva of the left eye (OS). Samples of AH and tears were collected on day 0, and days 2, 7, 14 and 23 post-injection for determination of voriconazole concentrations. At each of these timepoints, tears were collected via direct contact with the lacrimal lake (cul-de-sac) using plain clean glass microcapillary tubes (Fischer Scientific, Pittsburgh, PA). The tear fluid was placed in Eppendorf Tubes® for storage. Aqueous humor samples were collected immediately after and following eyelid blocks as described above. The conjunctival fornices were irrigated with 0.5% povidone-iodine solution. The bulbar conjunctiva was fixated with 0.3 colibri forceps and a 30 gauge needle was advanced through the limbus into the anterior chamber from the 1 o’clock (OS) position and 0.3–0.5 mL of AH was aspirated. Immediately following sample collection, a drop of moxifloxacin 0.5% (Vigamox®, Alcon, Fort Worth, TX) was applied topically. Following the procedure each horse received a single dose of flunixin meglumine (Banamine®, Merck, Germany) at a dose of 1.1 mg/kg IV. Tears and AH samples were stored at − 80 °C until determination of voriconazole concentrations.

Every horse underwent a complete ophthalmic examination prior to the treatment (day 0), and on days 2, 7, 14 and 23 post injection. Ocular inflammatory changes were given a score following a modified Hackett-McDonald scoring system [30]. This system records the ocular findings of slit lamp examinations, and has been used in toxicology studies evaluating ophthalmic medications [30]. The modified Hackett-McDonald scoring system used evaluates conjunctival congestion (grades 0 to 3), swelling (grades 0 to 4) and discharge (grades 0 to 3), as well as corneal cloudiness (grades 0 to 4), corneal neovascularization (grades 0 to 2), fluorescein staining intensity (grades 0 to 4), aqueous flare (grades 0 to 3), iris congestion (grades 0 to 4) [30]. Following ophthalmic examination, each category was given a score (0 if no changes were observed), and the values were compared within and between timepoints. Horses were also observed twice daily for signs of ocular pain (blepharospasm, blepharoedema, epiphora) for the duration of the study.

Following each of the treatments, the horses were stalled for 24 h, and then transferred to a pasture.

### Aim 2: Voriconazole ocular tissue concentrations and distribution

For the second aim, 6 horses (3 geldings and 3 mares), euthanized due to reasons unrelated to this study, were included. Ages ranged from 8 to 17 years, and breeds included 3 American Quarter Horses, 2 Tennessee Walking Horses and 1 Warmblood.

Three horses received 2 SCo injections of 0.3 mL each of 1.7% voriconazole-thermogel in the dorsal bulbar conjunctiva of each eye. The first injection was applied OS 48 h prior to euthanasia, and the second one OD 2 h before euthanasia. The remaining 3 horses received 0.3 mL of 1.7% voriconazole-thermogel SCo injection OS, and 0.3 mL of thermogel alone by SCo injection OD, both 7 days prior to euthanasia (Fig. 5). No randomization for treatment order was used.

**Fig. 6:**
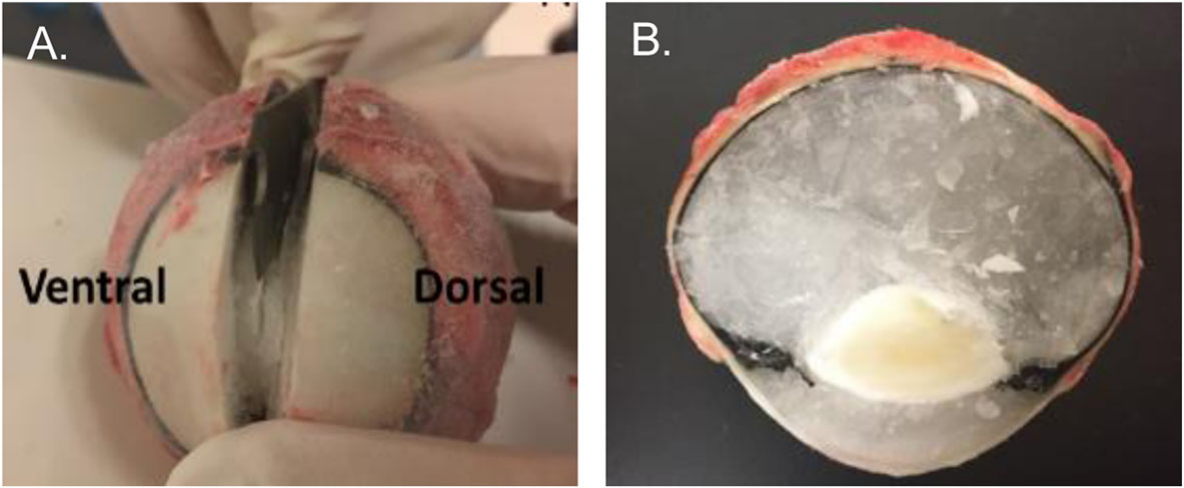
**a** Splitting of the frozen globe into dorsal and ventral segments. **b** Cross-section of a split frozen globe prior to dissection

Horses were euthanized by IV administration of pentobarbital sodium (78 mg/kg). Immediately after euthanasia both eyes were enucleated using a transpalpebral technique. Surrounding ocular tissues were removed and the whole globe was snap frozen in liquid nitrogen, followed by storage at − 80 °C until dissection was performed. The right eyes of the horses receiving thermogel alone (no drug) 7 days prior to euthanasia were fixed in 10% formalin solution for histological analysis. Histological analysis of the eyes were performed by a board-certified specialist in veterinary pathology at the Comparative Ocular Pathology Laboratory of Wisconsin. The frozen eyes were cut in half along the horizontal axis into dorsal and ventral segments using a microtome blade (Accu-Edge®) (Fig. 6a, b) [65]. Dissection was started on the dorsal segment and the ventral segment was stored in a − 20 °C freezer until use. The different tissues were individually dissected over a cooled tile, weighed, and immediately stored at − 80 °C until measurement of voriconazole concentrations.

### Animals

All animals involved in the study were part of the Auburn University teaching and research herd. A priori power calculations were performed using GPower v.3.1.9.2 to estimate the number of horses required for the study. Using a type 1 error of 0.05, a type 2 error of 0.8 and an estimated effect size of 1.4 (based on the mean and standard deviation of previous studies measuring voriconazole in AH [4, 54]), it was determined that 5–7 horses were needed per group. A total of 12 horses were included in the study, 6 for the first aim and 6 for the second aim. Use of animals in this study adhered to the ARVO Statement for the Use of Animals in Ophthalmic and Visual Research and was approved and monitored by the Auburn University Institutional Animal Care and Use Committee (protocols 2018–3232, 2017–3170, 2015–2663, and 2014–2491). Horses were determined to be healthy for inclusion in the study based on a normal physical examination, CBC and SBA. All the horses underwent a complete ophthalmic examination performed by a board-certified specialist in veterinary ophthalmology (EMA or RJM) and were determined to be free of ocular abnormalities. Complete ophthalmic examination included Schirmer tear test (STT, Schering-Plough, Charlotte, NC), slit lamp examination (Kowa SL-14, Tokyo, Japan), tonometry (TonoVet, iCare, Finland), indirect ophthalmoscopy (Keeler, Lombart Instruments, Norfolk, VA), and external fluorescein dye application (Akorn Inc., Buffalo Grove, IL). After pupillary dilation with tropicamide 1% (Akorn Inc., Buffalo Grove, IL), fundus images were obtained with a retina camera (Optibrand Clearview, Optibrand Ltd., Fort Collins CO). None of the horses received any systemic or ophthalmic medication for at least 60 days prior to their inclusion in this study. All studies were carried at the Auburn University Large Animal Teaching Hospital facilities. Grass hay and water were provided ad libitum. Horses involved in this study were housed in individual stalls, as well as in their original paddocks with the rest of the herd.

### Preparation of the Voriconazole-Thermogel

The thermogel was selected and prepared based on a previous study by our group [24]. Briefly, a combination of the thermogels PEG-PLGA-PEG AK19 (MW 1500–1500-1500, 1:1 lactide:glycolide) and AK24 (MW 1100–1000-1100, 3:1 lactide:glycolide) (Akina Inc., West Lafayette, IN) in a 1:1 ratio was used in order to reach a gelation temperature between 33.3 °C–35.3 °C (temperature of the SCo space in horses) [24]. The thermogel was stored at 4 °C thereafter, and 24 h before injection, both the AK19 and AK24 solutions were mixed in a single container.

Voriconazole powder USP (U.S. Pharmacopeia, Rockville, MD) was weighed and added to the liquid thermogel in an amount necessary to create a 1.7% suspension. All thermogel preparation was performed under a sterile products laminar flow hood in a US Pharmacopeal Convention (USP) 797 sterile products compounding room. Prior to injecting the voriconazole-thermogel, gelation was tested by immersing tubes containing the suspension into a water bath set at 34 °C. The thermogel was confirmed to go from liquid to gel once at 34 °C and back to liquid when stored at 4 °C for up to 3 weeks.

### Preparation of Voriconazole 1% solution

Voriconazole for IV use in humans containing beta cyclodextran (Sandoz, Princeton, NJ) was used for topical ophthalmic application [4]. A 1% solution was prepared by reconstitution of the powder with 19 mL of sterile water. The compounding was performed by the Auburn University Veterinary Pharmacy under USP 797 sterile compendium guidelines. The 1% voriconazole was prepared the day prior to utilization, and was stored in the refrigerator at 4 °C for the 48 h duration of the treatment.

### Subconjunctival injection of Voriconazole-Thermogel

The liquid thermogel was vortexed, and then 0.3 mL of the 1.7% (5 mg total) voriconazole-thermogel were drawn into a tuberculin syringe and stored on ice to maintain the liquid state. The suspension was mixed by rocking in the syringe just prior to injection. The injection was performed using a 30 gauge, ½ inch needle, and in all cases a well-defined gel deposit was observed immediately following the injection in the SCo space.

For the injection, the horses were placed in the stocks and sedated with detomidine hydrochloride (0.01 mg/kg, IV) (Dormosedan®, Zoetis Kalamazoo, MI). Auriculo-palpebral and supraorbital nerve blocks were performed by subcutaneous injection of 1 mL of 2% lidocaine hydrochloride (Hospira, Inc., Lake Forest, IL). Local anesthesia using proparacaine hydrochloride 0.5% (Akorn, Lake Forest, IL) was applied topically to the injection site in the dorsal bulbar conjunctiva.

### Determination of Voriconazole concentrations

Phosphate-buffered saline samples were analyzed by reverse phase high performance liquid chromatography (HPLC) as described elsewhere [34, 54, 66]. The HPLC system (Agilent 1200 series) encompassed pumps, autosampler, UV and visible light absorption detector, column (Thermo BetaBasic-18, 4.6 mm × 15 cm, 5 μ; Bellefonte, PA, USA), and computer interface. The flow rate was 0.75 mL/min and mobile phase was 35% 0.1 M N, N, N′, N′-tetramethylenediamine (Fisher Scientific, Inc., Waltham, MA, USA) and methanol (Fisher Scientific, Inc.). An injection volume of 100 μL was used. UV detection was at 254 nm with voriconazole, and ketoconazole as the internal standard, resulting in retention times of 3.7 and 13.5 min respectively. Calibration standards for voriconazole ranged from 0.001 to 10 μg/mL and were prepared in dissolution media as well as selected fluids. Plasma and tissue samples collected from horses that had not received voriconazole were used as controls and for preparation of standard curves. After adding internal standard tissues were minced and extracted with 4 mL of ethyl acetate. The lower limit of detection and quantification for voriconazole in plasma and tissues was 0.001 μg/mL(μg/g) and 0.005 μg/mL(μg/g) respectively. Values of 1.96 and 5.01% were noted for intra- and interday variations respectively. Extraction efficiency was greater than 89% for drug and internal standard.

### Data analysis

Data were evaluated for normality using a Shapiro-Wilk test and non-parametric analysis used when appropriate. Scores from the modified Hackett-McDonald scoring system were compared among days and treatments with Friedman’s test and Dunn’s post hoc test. Non-continuous score data were presented as median and range. Voriconazole concentrations in tears at day 2 between the Vori-Top and Vori-Gel groups were compared using a Wilcoxon matched pairs signed rank test. Pooled voriconazole concentrations were compared between dorsal and ventral sections of the frozen eyes and between tissues of the anterior segment (cornea, AH, iris-ciliary body, sclera and lens) and posterior segment (vitreous, retina, choroid) using a Kruskal Wallis test and Dunn’s post hoc test. Sclera was included as part of the anterior segment since the injection was applied in the anterior scleral surface. Voriconazole concentrations between individual tissues and timepoints were compared using a repeated measures 2-way ANOVA with Tukey’s multiple comparison test. Significance was set at *p* ≤ 0.05. Data were analyzed using commercial software (GraphPad Prism v.6).

## Data Availability

The datasets analyzed are available from the corresponding author on reasonable request.

## Abbreviations

SCo: Subconjunctival
PLGA-PEG-PLGA: Poly (DL-lactide-co-glycolide-b-ethylene glycol-b-DL-lactide-co-glycolide)
AH: Aqueous humor
OD: Right eye
OS: Left eye
MIC: Minimum inhibitory concentration
CBC: Complete blood count
SBA: Serum biochemical analysis
SD: Standard deviation
HPLC: High performance liquid chromatography
